# Supplementary material for: A Likelihood Approach for Real-Time Calibration of Stochastic Compartmental Epidemic Models
Source: PLoS Comput Biol. 2017 Jan 17;13(1):e1005257. doi: 10.1371/journal.pcbi.1005257 (PMC5240920; doi:10.1371/journal.pcbi.1005257)
Supplement: S1 File — (TAR.GZ) [file pcbi.1005257.s014.tar.gz › HSPH_Online-SI-Revision/MSS10/n100-mild/n100-mild_NEW_table4.pdf]

1	R0	{1.24125, 3.38982, 0.1}	{1.22937, 3.13594, 0.21}	{{1.22544, 3.07157, 0.}, {1.25474, 3.26445, 0.}}	{1.25987, 9.29017, 296.}	inf	918.	876.596	{745.107, 1006.16}	10.107	0.0457065	newly	1628.	1643.54	{1443., 1821.}	8.13636	0.0354131	Reff	0.854936	0.847258	{0.843776, 0.861423}	1.15141	ar	34926.	35083.5	{34276., 36035.}	1.60594
2	R0	{1.29554, 4.51878, 0.1}	{1.31639, 4.25307, 2.}	{{1.31748, 4.25564, 2.}, {1.31748, 4.25564, 2.}}	{1.60902, 5.88009, 1900.}	inf	1374.	1340.93	{1236.95, 1439.39}	4.72201	0.0210276	newly	1875.	1891.27	{1738., 2038.}	4.9424	0.0213148	Reff	0.799646	0.812947	{0.812044, 0.814834}	1.6634	ar	42828.	43040.3	{42457., 43635.}	0.926497
3	R0	{1.2443, 3.64292, 0.1}	{1.25386, 3.25866, 0.}	{{1.25474, 3.26445, 0.}, {1.25474, 3.26445, 0.}}	{0.859266, 10.5481, 100.}	inf	748.	712.06	{609.042, 800.637}	9.25429	0.0422788	newly	1275.	1277.84	{1082., 1447.}	9.35529	0.0409026	Reff	0.825668	0.832462	{0.831457, 0.834273}	0.905396	ar	37087.	36653.8	{35973., 37263.}	1.48481
4	R0	{1.38465, 3.43331, 0.1}	{1.37877, 3.32668, 4.9}	{{1.37974, 3.3318, 5.}, {1.37974, 3.3318, 5.}}	{0.424696, 3.1058, 4800.}	inf	741.	615.541	{538.063, 692.655}	17.0745	0.0832089	newly	1149.	981.09	{852., 1114.}	15.04	0.0726843	Reff	0.722441	0.721102	{0.720195, 0.722649}	0.199207	ar	49511.	49215.	{48865., 49535.}	0.670619
5	R0	{1.25409, 4.1068, 0.1}	{1.25667, 3.32453, 0.09}	{{1.25474, 3.26445, 0.}, {1.25474, 3.26445, 0.}}	{0.205475, 19.0481, 184.}	inf	1115.	922.907	{815.887, 1018.21}	17.7417	0.0863048	newly	1641.	1614.92	{1413., 1787.}	6.92505	0.0306783	Reff	0.822708	0.826808	{0.824642, 0.827417}	0.498391	ar	38571.	37932.	{37233., 38636.}	2.0323
6	R0	{1.28821, 4.2016, 0.1}	{1.28596, 3.98513, 0.}	{{1.28596, 3.98513, 0.}, {1.28596, 3.98513, 0.}}	{0.174748, 5.15203, 100.}	inf	1013.	887.202	{801.698, 988.088}	13.0916	0.0620318	newly	1399.	1304.76	{1169., 1464.}	9.13796	0.0419737	Reff	0.800326	0.800545	{0.799071, 0.801631}	0.101336	ar	41468.	40881.2	{40274., 41397.}	1.54495
7	R0	{1.24369, 2.56007, 0.1}	{1.24751, 2.5364, 0.}	{{1.24751, 2.5364, 0.}, {1.24751, 2.5364, 0.}}	{0.307391, 0.924408, 100.}	inf	442.	457.079	{358.783, 570.778}	15.7495	0.0667218	newly	924.	941.4	{731., 1151.}	15.3203	0.0661925	Reff	0.781634	0.783849	{0.782412, 0.785047}	0.288612	ar	38603.	38707.6	{38201., 39199.}	0.771857
8	R0	{1.232, 3.53942, 0.1}	{1.22544, 3.07157, 0.}	{{1.22544, 3.07157, 0.}, {1.22544, 3.07157, 0.}}	{0.532715, 13.2182, 100.}	inf	649.	527.508	{423.138, 623.666}	19.2628	0.0977806	newly	1057.	994.05	{780., 1194.}	12.7067	0.0599105	Reff	0.831846	0.828904	{0.827682, 0.830145}	0.353739	ar	34555.	34631.3	{33885., 35255.}	1.14745
9	R0	{1.36262, 3.85631, 0.1}	{1.36035, 3.22945, 3.}	{{1.36035, 3.22945, 3.}, {1.36035, 3.22945, 3.}}	{0.166696, 16.2554, 2900.}	inf	951.	755.85	{633.488, 846.728}	20.5205	0.102021	newly	1368.	1257.	{1073., 1418.}	10.0015	0.0468893	Reff	0.74132	0.742739	{0.741396, 0.744247}	0.202401	ar	48410.	47454.7	{46953., 47875.}	1.97333
10	R0	{1.28702, 4.67968, 1.}	{1.28836, 4.02612, 0.36}	{{1.28596, 3.98513, 0.}, {1.29927, 4.21286, 2.}}	{0.23899, 13.9658, 100.}	inf	1183.	1023.74	{896.017, 1151.11}	13.642	0.065412	newly	1557.	1508.73	{1314., 1668.}	7.10405	0.03201	Reff	0.80682	0.809708	{0.806644, 0.815826}	0.368714	ar	41092.	40856.	{40158., 41384.}	1.05872
11	R0	{1.35752, 3.47129, 0.1}	{1.31492, 3.46008, 4.94}	{{1.31535, 3.47086, 5.}, {1.31535, 3.47086, 5.}}	{3.13803, 0.32292, 4840.}	inf	1161.	1034.97	{903.993, 1146.93}	11.3673	0.0538567	newly	1963.	1673.3	{1473., 1863.}	14.8803	0.0716984	Reff	0.799932	0.776487	{0.774966, 0.77823}	2.9309	ar	44669.	44145.9	{43586., 44666.}	1.3024
12	R0	{1.35929, 5.83647, 0.1}	{1.34002, 5.27199, 5.}	{{1.34002, 5.27199, 5.}, {1.34002, 5.27199, 5.}}	{1.41758, 9.67161, 4900.}	inf	1940.	1656.62	{1577.09, 1739.64}	14.6074	0.0689419	newly	2036.	1930.33	{1801., 2025.}	5.52505	0.025033	Reff	0.813535	0.8058	{0.804651, 0.806832}	0.950804	ar	46715.	45342.1	{44746., 45977.}	2.93897
13	R0	{1.28019, 3.29908, 0.1}	{1.30025, 3.11521, 3.}	{{1.30094, 3.11158, 3.}, {1.30094, 3.11158, 3.}}	{1.60982, 5.57339, 2900.}	inf	878.	800.819	{667.141, 920.459}	11.2512	0.0533906	newly	1478.	1447.75	{1221., 1667.}	9.89919	0.044363	Reff	0.791618	0.805029	{0.803789, 0.807115}	1.74064	ar	40941.	40948.2	{40302., 41610.}	0.997802
14	R0	{1.36661, 3.48318, 0.1}	{1.36132, 3.23457, 3.1}	{{1.36035, 3.22945, 3.}, {1.36035, 3.22945, 3.}}	{0.483299, 7.13745, 3000.}	inf	608.	550.874	{464.766, 630.29}	11.5205	0.0548742	newly	929.	895.15	{737., 1037.}	9.89343	0.0451684	Reff	0.726053	0.724019	{0.722278, 0.724989}	0.389405	ar	48672.	48180.	{47787., 48527.}	1.02698
15	R0	{1.35865, 3.84123, 0.1}	{1.36035, 3.22945, 3.}	{{1.36035, 3.22945, 3.}, {1.36035, 3.22945, 3.}}	{0.125019, 15.9266, 2900.}	inf	1150.	871.901	{754.964, 987.633}	24.1825	0.122857	newly	1651.	1441.74	{1239., 1649.}	13.0406	0.063086	Reff	0.73825	0.742956	{0.741381, 0.744544}	0.637463	ar	48598.	47684.4	{47190., 48073.}	1.87995
16	R0	{1.22879, 3.98329, 0.1}	{1.22474, 3.09669, 0.05}	{{1.22544, 3.07157, 0.}, {1.22544, 3.07157, 0.}}	{0.329201, 22.258, 140.}	inf	1035.	781.117	{658.992, 897.51}	24.5507	0.125427	newly	1786.	1534.14	{1288., 1751.}	14.5823	0.0711655	Reff	0.876398	0.876623	{0.875609, 0.878603}	0.179664	ar	34405.	32757.8	{31709., 33683.}	4.79224
17	R0	{1.34925, 3.64524, 0.1}	{1.36035, 3.22945, 3.}	{{1.36035, 3.22945, 3.}, {1.36035, 3.22945, 3.}}	{0.822573, 11.4063, 2900.}	inf	1239.	990.222	{862.807, 1117.89}	20.079	0.0993657	newly	1851.	1671.81	{1454., 1900.}	10.9719	0.0518295	Reff	0.759911	0.769546	{0.767723, 0.771228}	1.26792	ar	46871.	46338.1	{45808., 46856.}	1.18303
18	R0	{1.33054, 2.25275, 0.1}	{1.31246, 2.23442, 4.}	{{1.31246, 2.23442, 4.}, {1.31246, 2.23442, 4.}}	{1.35899, 0.813541, 3900.}	inf	312.	336.868	{264.869, 412.745}	17.7007	0.0732253	newly	665.	723.77	{547., 898.}	18.1338	0.0744683	Reff	0.750025	0.739506	{0.738505, 0.740441}	1.40251	ar	44336.	44587.9	{44190., 44930.}	0.693635
19	R0	{1.28394, 3.69859, 0.1}	{1.26746, 3.5533, 0.06}	{{1.25474, 3.26445, 0.}, {1.28596, 3.98513, 0.}}	{1.40907, 10.1262, 156.}	inf	963.	945.178	{764.733, 1139.98}	11.9256	0.0530227	newly	1523.	1542.16	{1341., 1750.}	7.89757	0.0341746	Reff	0.824444	0.814073	{0.806132, 0.824988}	1.2907	ar	39558.	39409.2	{38496., 40467.}	1.75785
20	R0	{1.2883, 2.26493, 0.1}	{1.30627, 2.18661, 4.}	{{1.27121, 1.91565, 4.}, {1.31246, 2.23442, 4.}}	{1.79285, 3.4581, 3900.}	inf	531.	548.892	{396.256, 688.161}	18.307	0.079001	newly	1246.	1277.35	{924., 1599.}	17.6974	0.07678	Reff	0.78634	0.797069	{0.777446, 0.802573}	1.72458	ar	40768.	40968.9	{40203., 41651.}	1.18365
21	R0	{1.34098, 2.78621, 0.1}	{1.31246, 2.23442, 4.}	{{1.31246, 2.23442, 4.}, {1.31246, 2.23442, 4.}}	{2.12695, 19.8042, 3900.}	inf	549.	399.67	{300.631, 509.127}	27.6105	0.148588	newly	1041.	851.18	{622., 1066.}	20.292	0.104229	Reff	0.763192	0.748919	{0.74746, 0.750155}	1.87015	ar	44605.	44048.1	{43619., 44476.}	1.29746
22	R0	{1.33783, 5.95911, 0.1}	{1.31571, 4.24951, 1.98}	{{1.31748, 4.25564, 2.}, {1.31748, 4.25564, 2.}}	{1.6537, 28.6888, 1882.}	inf	1990.	1411.01	{1291.5, 1509.4}	29.095	0.150056	newly	2166.	1998.19	{1831., 2163.}	7.94506	0.0366268	Reff	0.826485	0.820434	{0.819256, 0.822879}	0.732036	ar	45418.	42776.7	{42167., 43406.}	5.81547
23	R0	{1.33359, 3.3069, 0.1}	{1.36035, 3.22945, 3.}	{{1.36035, 3.22945, 3.}, {1.36035, 3.22945, 3.}}	{2.00651, 2.342, 2900.}	inf	1009.	929.076	{805.42, 1039.78}	9.89338	0.0463751	newly	1653.	1575.42	{1341., 1747.}	8.28433	0.037955	Reff	0.751291	0.767453	{0.765916, 0.769106}	2.15122	ar	46245.	46382.5	{45845., 46820.}	0.695729
24	R0	{1.30588, 2.56123, 0.1}	{1.31246, 2.23442, 4.}	{{1.31246, 2.23442, 4.}, {1.31246, 2.23442, 4.}}	{0.503724, 12.7598, 3900.}	inf	372.	341.496	{214.845, 440.129}	19.459	0.0971067	newly	702.	762.43	{479., 988.}	24.839	0.105665	Reff	0.762347	0.766587	{0.765068, 0.768032}	0.556241	ar	42383.	42666.6	{42108., 43150.}	0.954392
25	R0	{1.29813, 4.51849, 0.1}	{1.29907, 4.19863, 1.86}	{{1.29927, 4.21286, 2.}, {1.29927, 4.21286, 2.}}	{0.203684, 7.07886, 1774.}	inf	1528.	1443.74	{1299.3, 1573.5}	6.99822	0.0320193	newly	2037.	2057.13	{1856., 2251.}	5.53216	0.0238951	Reff	0.813343	0.815024	{0.813013, 0.816795}	0.28851	ar	43371.	42393.2	{41779., 43049.}	2.29946
26	R0	{1.33231, 3.99953, 0.1}	{1.31535, 3.47086, 5.}	{{1.31535, 3.47086, 5.}, {1.31535, 3.47086, 5.}}	{1.27275, 13.2183, 4900.}	inf	1265.	959.804	{876.167, 1044.06}	24.1261	0.121052	newly	1843.	1556.82	{1399., 1725.}	15.5279	0.0746004	Reff	0.789434	0.7834	{0.782179, 0.784496}	0.764238	ar	44733.	43559.5	{43043., 44024.}	2.62343
27	R0	{1.30555, 4.61445, 0.1}	{1.28716, 4.00563, 0.18}	{{1.28596, 3.98513, 0.}, {1.28596, 3.98513, 0.}}	{1.40881, 13.1938, 262.}	inf	1370.	1208.36	{1096.11, 1320.5}	11.9445	0.0562203	newly	1833.	1782.58	{1618., 1948.}	5.96399	0.02665	Reff	0.814154	0.804763	{0.802974, 0.806483}	1.15342	ar	42624.	41681.	{41060., 42263.}	2.23163
28	R0	{1.26819, 5.34969, 0.1}	{1.25325, 3.45542, 1.1}	{{1.25474, 3.26445, 0.}, {1.25656, 3.82585, 3.}}	{1.17816, 35.4089, 1128.}	inf	1478.	928.957	{748.605, 1093.37}	37.1477	0.206027	newly	1792.	1608.94	{1342., 1820.}	11.231	0.0540264	Reff	0.858552	0.855315	{0.854151, 0.858259}	0.392276	ar	38946.	36145.2	{34961., 37049.}	7.1915
29	R0	{1.19925, 3.00394, 0.1}	{1.19657, 2.14148, 2.64}	{{1.19264, 2.01465, 0.}, {1.22544, 3.07157, 3.}}	{0.747123, 29.2512, 2564.}	inf	570.	455.844	{282.114, 647.592}	25.8856	0.142285	newly	1093.	1195.26	{787., 1554.}	23.5462	0.101016	Reff	0.866182	0.865608	{0.861549, 0.882172}	0.561527	ar	31727.	30257.8	{29064., 31707.}	5.10827
30	R0	{1.35749, 3.75483, 0.1}	{1.36112, 3.23355, 3.08}	{{1.36035, 3.22945, 3.}, {1.36035, 3.22945, 3.}}	{0.267713, 13.883, 2980.}	inf	1015.	811.845	{703.92, 914.394}	20.0153	0.0988091	newly	1413.	1326.57	{1153., 1496.}	9.12456	0.0420412	Reff	0.732529	0.737254	{0.735448, 0.738371}	0.645123	ar	48244.	47918.1	{47517., 48352.}	0.790295
31	R0	{1.27131, 4.2161, 0.1}	{1.25474, 3.26445, 0.}	{{1.25474, 3.26445, 0.}, {1.25474, 3.26445, 0.}}	{1.30345, 22.5719, 100.}	inf	1215.	952.629	{842.654, 1046.64}	21.5943	0.107461	newly	1803.	1707.9	{1486., 1901.}	7.89906	0.0366063	Reff	0.843883	0.836175	{0.834928, 0.837526}	0.913339	ar	38299.	37436.5	{36693., 38141.}	2.26189
32	R0	{1.31827, 6.18119, 0.1}	{1.29901, 4.20831, 1.96}	{{1.29927, 4.21286, 2.}, {1.29927, 4.21286, 2.}}	{1.46118, 31.9175, 1864.}	inf	1873.	1197.95	{1100.02, 1289.15}	36.0409	0.194879	newly	1902.	1709.18	{1567., 1865.}	10.3807	0.0483655	Reff	0.818804	0.815609	{0.814423, 0.817025}	0.390243	ar	45262.	41554.8	{41085., 42127.}	8.19047
33	R0	{1.20398, 1.35167, 0.1}	{1.2663, 1.35135, 3.}	{{1.2663, 1.35135, 3.}, {1.2663, 1.35135, 3.}}	{5.17577, 0.0236939, 2900.}	inf	90.	127.194	{29.9046, 233.86}	78.4473	0.325073	newly	298.	458.19	{88., 810.}	86.6812	0.341642	Reff	0.806895	0.848187	{0.846825, 0.849416}	5.1174	ar	33329.	33730.1	{33018., 34387.}	1.56977
34	R0	{1.32776, 3.146, 0.1}	{1.31023, 3.04996, 5.}	{{1.31023, 3.04996, 5.}, {1.31023, 3.04996, 5.}}	{1.32062, 3.0527, 4900.}	inf	466.	453.806	{351.987, 550.466}	12.5	0.0567758	newly	799.	776.41	{609., 922.}	12.5169	0.0569074	Reff	0.745949	0.736257	{0.734977, 0.73751}	1.2992	ar	44872.	45009.7	{44536., 45353.}	0.624911
35	R0	{1.279, 1.11336, 0.1}	{1.30222, 1.35944, 3.}	{{1.30222, 1.35944, 3.}, {1.30222, 1.35944, 3.}}	{1.81542, 22.1022, 2900.}	inf	15.	39.6689	{7.2849, 82.7095}	183.776	0.431348	newly	47.	120.96	{19., 251.}	179.149	0.437167	Reff	0.754597	0.767975	{0.767384, 0.768381}	1.77285	ar	41033.	41158.5	{41012., 41356.}	0.327566
36	R0	{1.20029, 1.94717, 0.1}	{1.19264, 2.01465, 3.}	{{1.19264, 2.01465, 3.}, {1.19264, 2.01465, 3.}}	{0.637407, 3.46562, 2900.}	inf	338.	297.674	{158.422, 418.057}	25.2187	0.132348	newly	910.	768.	{411., 1113.}	26.0593	0.140863	Reff	0.82688	0.82209	{0.820651, 0.823713}	0.579244	ar	33234.	32355.4	{31543., 33089.}	2.70723
37	R0	{1.33216, 3.69685, 0.1}	{1.31341, 3.31995, 4.98}	{{1.31023, 3.04996, 5.}, {1.31535, 3.47086, 5.}}	{1.40717, 10.1951, 4880.}	inf	945.	827.257	{676.221, 943.946}	13.5061	0.0658979	newly	1507.	1362.1	{1132., 1532.}	10.8281	0.0516329	Reff	0.770335	0.761039	{0.759334, 0.762472}	1.20668	ar	45235.	44423.4	{43840., 44944.}	1.80734
38	R0	{1.25434, 2.04314, 0.1}	{1.27121, 1.91565, 4.}	{{1.27121, 1.91565, 4.}, {1.27121, 1.91565, 4.}}	{1.34522, 6.24007, 3900.}	inf	246.	216.664	{101.012, 331.634}	31.904	0.191407	newly	612.	544.75	{249., 854.}	32.9788	0.193102	Reff	0.777829	0.788665	{0.78717, 0.790033}	1.39317	ar	38662.	38723.4	{38189., 39289.}	0.937277
39	R0	{1.27466, 3.0741, 0.1}	{1.25553, 2.62268, 0.45}	{{1.24751, 2.5364, 0.}, {1.30094, 3.11158, 3.}}	{2.11956, 15.0504, 520.}	inf	499.	421.7	{301.819, 531.024}	18.8477	0.0970992	newly	937.	837.53	{607., 1038.}	16.2337	0.0807776	Reff	0.783495	0.772698	{0.76666, 0.799003}	1.99131	ar	40181.	39820.2	{39191., 40398.}	1.23041
40	R0	{1.33045, 2.68154, 0.1}	{1.31246, 2.23442, 4.}	{{1.31246, 2.23442, 4.}, {1.31246, 2.23442, 4.}}	{1.35232, 16.6739, 3900.}	inf	318.	262.659	{186.59, 336.615}	20.462	0.107217	newly	616.	546.01	{389., 697.}	18.2419	0.0920664	Reff	0.729978	0.720833	{0.719837, 0.721816}	1.25282	ar	45951.	45750.3	{45436., 46061.}	0.542621
41	R0	{1.37003, 2.83984, 0.1}	{1.37363, 2.81014, 3.}	{{1.37363, 2.81014, 3.}, {1.37363, 2.81014, 3.}}	{0.263126, 1.04578, 2900.}	inf	516.	516.827	{419.262, 622.859}	12.2816	0.0539802	newly	894.	924.58	{745., 1117.}	13.4966	0.0574069	Reff	0.718704	0.720584	{0.719079, 0.721914}	0.270079	ar	48926.	48813.1	{48427., 49169.}	0.528226
42	R0	{1.3003, 2.94885, 0.1}	{1.31246, 2.23442, 4.}	{{1.31246, 2.23442, 4.}, {1.31246, 2.23442, 4.}}	{0.935018, 24.2273, 3900.}	inf	576.	403.395	{303.49, 526.618}	29.9741	0.165015	newly	1090.	876.91	{637., 1155.}	22.2321	0.116361	Reff	0.746177	0.755419	{0.753802, 0.756716}	1.23862	ar	44237.	43597.	{43120., 44175.}	1.48887
43	R0	{1.33756, 2.92653, 0.1}	{1.36141, 3.19591, 3.}	{{1.36035, 3.22945, 3.}, {1.36035, 3.22945, 3.}}	{1.78321, 9.841, 2900.}	inf	776.	911.146	{754.124, 1036.33}	19.3504	0.0757572	newly	1372.	1512.49	{1310., 1704.}	12.9949	0.0525763	Reff	0.730161	0.741343	{0.738998, 0.743137}	1.53147	ar	47342.	47921.8	{47349., 48362.}	1.30455
44	R0	{1.35801, 2.92276, 0.1}	{1.37363, 2.81014, 3.}	{{1.37363, 2.81014, 3.}, {1.37363, 2.81014, 3.}}	{1.15057, 3.85315, 2900.}	inf	436.	461.722	{361.255, 554.356}	14.5678	0.0611413	newly	782.	834.23	{645., 1016.}	14.922	0.0618984	Reff	0.722081	0.730036	{0.728755, 0.731403}	1.10164	ar	47940.	48052.9	{47665., 48428.}	0.524113
45	R0	{1.31342, 4.02649, 0.1}	{1.31748, 4.25564, 2.}	{{1.31748, 4.25564, 2.}, {1.31748, 4.25564, 2.}}	{0.308945, 5.69102, 1900.}	inf	1233.	1371.53	{1258.59, 1494.07}	11.4125	0.0461676	newly	1772.	1907.64	{1762., 2084.}	8.3702	0.0343402	Reff	0.7963	0.796935	{0.795306, 0.798395}	0.133912	ar	42995.	44008.5	{43504., 44480.}	2.35725
46	R0	{1.37919, 3.75889, 0.1}	{1.38824, 4.0768, 4.15}	{{1.37974, 3.3318, 4.}, {1.40116, 4.25615, 5.}}	{0.710699, 12.8842, 4050.}	inf	1312.	1540.33	{1201.22, 1721.44}	21.1703	0.0836644	newly	1998.	2131.95	{1926., 2295.}	9.30881	0.0388376	Reff	0.747438	0.749163	{0.746734, 0.753609}	0.310066	ar	49157.	49968.2	{48807., 50637.}	1.99705
47	R0	{1.37458, 3.56117, 0.1}	{1.36054, 3.23048, 3.02}	{{1.36035, 3.22945, 3.}, {1.36035, 3.22945, 3.}}	{1.02873, 9.28611, 2920.}	inf	838.	747.758	{653.173, 846.153}	11.7418	0.0555449	newly	1272.	1222.26	{1052., 1389.}	8.52673	0.0385931	Reff	0.732981	0.726723	{0.725281, 0.727907}	0.860915	ar	48478.	48460.9	{48140., 48824.}	0.462581
48	R0	{1.33393, 3.78296, 0.1}	{1.31535, 3.47086, 5.}	{{1.31535, 3.47086, 5.}, {1.31535, 3.47086, 5.}}	{1.39265, 8.25018, 4900.}	inf	1093.	925.716	{812.639, 1027.23}	15.8098	0.0763752	newly	1686.	1485.34	{1287., 1648.}	12.8636	0.0612341	Reff	0.779095	0.770445	{0.769028, 0.771917}	1.11023	ar	44885.	44171.4	{43613., 44704.}	1.65322
49	R0	{1.24918, 2.39046, 0.1}	{1.27102, 1.89307, 3.96}	{{1.27121, 1.91565, 4.}, {1.27121, 1.91565, 4.}}	{1.7481, 20.8071, 3860.}	inf	342.	305.201	{184.613, 458.27}	25.1785	0.128171	newly	806.	835.39	{494., 1250.}	26.3883	0.117732	Reff	0.817189	0.831941	{0.830076, 0.83358}	1.80531	ar	35969.	35960.8	{35157., 36799.}	1.34869
50	R0	{1.28281, 2.3032, 0.1}	{1.27121, 1.91565, 4.}	{{1.27121, 1.91565, 4.}, {1.27121, 1.91565, 4.}}	{0.90398, 16.8267, 3900.}	inf	333.	272.093	{145.656, 379.612}	25.2766	0.141144	newly	744.	672.88	{378., 945.}	23.4382	0.122063	Reff	0.782989	0.776685	{0.7753, 0.778236}	0.805095	ar	40223.	39801.	{39291., 40272.}	1.17162
